# Flavoring Agents in E-cigarette Liquids: A Comprehensive Analysis of Multiple Health Risks

**DOI:** 10.7759/cureus.48995

**Published:** 2023-11-18

**Authors:** Jaspreet Sachdeva, Anisha Karunananthan, Jianru Shi, Wangde Dai, Michael T Kleinman, David Herman, Robert A Kloner

**Affiliations:** 1 Cardiovascular Sciences, Huntington Medical Research Institutes, Pasadena, USA; 2 Environmental and Occupational Health, College of Health Sciences, University of California, Irvine, USA

**Keywords:** epithelial barrier dysfunction, endothelial dysfunction, neuro-behavioral changes, cardiac electrophysiological alterations, inflammation, vaping, e-cigarette, flavor

## Abstract

The availability of a wide range of flavored e-cigarettes is one of the primary reasons for vaping initiation and persistent use among adolescents and young people. This plethora of flavors available on the market are crafted using different flavoring agents such as cinnamaldehyde, vanillin, benzaldehyde, ethyl maltol, menthol, and dimethylpyrazine. Recent studies have brought to light the potential risks associated with e-cigarette flavoring agents and their effects on various organ systems, both with and without nicotine. Research has demonstrated that flavoring agents can induce inflammation, endothelial dysfunction, epithelial barrier disruption, oxidative stress, DNA damage, electrophysiological alterations, immunomodulatory effects, and behavioral changes, even independently of nicotine. Notably, these negative outcomes adversely affect cardiovascular system by reducing cell viability, decreasing endothelial nitric oxide synthase, nitric oxide bioavailability, soluble guanylyl cyclase activity and cyclic guanosine monophosphate accumulation, impairing endothelial proliferation and tube formation, and altering vasoreactivity resulting in vascular dysfunction. In the heart, these agents decrease parasympathetic activity, induce depolarization of resting membrane potential, loss of rhythmicity, increase isovolumic relaxation time, and change in ventricular repolarization and ventricular tachyarrhythmias. It is found that the specific response elicited by flavoring agents in different organ systems varies depending on the flavor used, the concentration of the flavoring agent, and the duration of exposure. However, the literature on the effects of flavoring agents is currently limited, emphasizing the need for more preclinical and randomized clinical trials to gain a deeper understanding and provide further evidence of the harmful effects of flavored e-cigarette use. In summary, recent research suggests that flavoring agents themselves can have detrimental effects on the body. To fully comprehend these effects, additional preclinical and clinical studies are needed to explore the risks associated with flavored e-cigarette usage.

## Introduction and background

In the United States, adolescents and young adults (18-24 years) have traded tobacco-based cigarettes for e-cigarettes (e-C) in the form of disposables, prefilled or refillable vape pods/cartridges, and tank or mod-type devices resulting in an explosion of electronic nicotine delivery system (ENDS) use [1]. According to the Centers for Disease Control and Prevention (CDC), more than 99% of e-C sold in the United States contain nicotine [2]. The doses of nicotine in the popular pods that are typically used by youths can be very high reaching nearly 6% (nicotine concentrations expressed in percentages are equivalent to mg/mL of e-liquid, e.g., 2.4% = 24 mg/mL, free base nicotine is used up to 12 mg/mL concentration and nicotine salts, i.e., free base nicotine + benzoic acid that lowers pH and removes bitter taste to achieve 20-59 mg/mL concentrations) [3,4]. In a 2022 National Youth Tobacco Survey report, the CDC and the United States Food and Drug Administration (FDA) reported that 2.55 million middle (3.3%) and high school (14.1%) students are current e-C users. Among current e-C users, 27.6% (46% of high school students and 20.8% of middle schoolers) vape daily, and 42.3% have been using e-C for 20 or more of the past 30 days. Surprisingly, the overall number of current e-C users who preferred flavored products is 84.9% with the highest to lowest preference for fruit flavors (69.1%), candy, dessert or other sweets (38.3%), mint (29.4%) and menthol 16.7% and a similar pattern of flavor preference was observed with each type of e-C device available [5]. Among different devices, disposables were used by the majority of e-C users 55.3% (45.8% middle school and 57.2% high school students). CDC and CDC Foundation-Truth Initiative analyzed retail scanner data to assess unit sales of e-C by product and flavor type in the United States. The unit sales of disposable products increased from 10.3% to 19.8%, whereas prefilled cartridges decreased from 89.4% to 80.2% from August 2019 to May 2020 [6]. This could be attributed to the growing popularity of disposable products with flavors and the availability of flavored products in the online marketplace.

A national online survey conducted in 2016 on e-C users aged 18-64 years demonstrated that flavors are reportedly a common reason for initiating vaping; in the age group of 18 to 24 years, the odds of initiation due to flavor additives were significantly (p ≤ 0.05) greater than for older users (35-64 years age group). Fruit and candy flavors are the most popular flavoring agents among current young adult users (18-24 years) and a high percentage (63%) prefer flavorings other than tobacco, which was widely chosen in the past [7,8]. Adolescents believe that fruit-flavored e-C are less harmful than those that are tobacco-flavored which has, in part, led to legislative oversight [9]. The FDA 2020 ban on the sales of some JUUL products (tobacco or menthol-flavored prefilled cartridges/pods) diverted users to other commercial devices such as disposable e-C and mod-style (refillable) devices. Fruit and sweet flavorings are still legal for prefilled or refillable cartridge/pod vape devices and other custom-made e-liquid mixes containing a plethora of flavors can be vaporized and inhaled [10]. In a recent study, Gravely et al. showed that the prevalence of fruit/other flavored cartridges use among ENDS users (18 years and older, 36.9% 18-24 years) increased from 7.9% to 12.4% and menthol/mint cartridges from 7.1% to 13% in the first five months post 2020 US FDA ENDS enforcement priority. Among the flavored cartridge users (prior to enforcement) 54.6% switched to a flavor and/or device excluded from the enforcement priority and the usage of non-tobacco flavored disposable devices increased from 5.9% to 10.8% in 2020 compared to 2018 [11]. El-Hellani et al. also reported that do-it-yourself (DIY) additives mixed with unflavored, or menthol/tobacco flavored e-liquids increase aerosol toxicant emissions and higher reactive oxygen species (ROS) emission with free-base nicotine; these toxicants are comparable to commercial flavored e-liquids [12]. In this review article, we aimed to provide a comprehensive overview of the impact of flavored e-cigarettes (e-C) on various organ systems based on relevant peer-reviewed research articles.

## Review

To identify studies specifically focused on e-C flavors, we conducted a thorough search on the National Library of Medicine's PubMed database from its inception to July 2023, using a combination of keywords such as "e-cigarette," "vaping," "flavors," "flavoring agents," "heart," "lung," "brain," "endothelial dysfunction," "organ system," "inflammation," and "metabolic." Our search yielded a total of 26 studies, with a subset of 10 studies focusing on flavors in a nicotine-free environment, comprising both in vitro and in vivo investigations that examined the effects of e-C flavors and/or their constituents, flavoring agents. Additionally, two human studies were included, revealing that e-C users found flavors to be pleasant and satisfying, potentially contributing to their increased usage. Importantly, none of the clinical trials reviewed indicated a link between flavored e-C use and addiction.

Flavoring agents

In this article, we have used both terms - flavor and/or flavoring agent based on whether the study was focused on individual flavoring agent (flavoring agents) or a combination of flavoring agents (flavor). Flavored e-liquids, including options like menthol, mint, cinnamon, fruit, and candy, are readily available in the market. They are formulated by blending a variety of flavoring agents, such as menthol, cinnamaldehyde, vanillin, ethyl maltol, and dimethylpyrazine (Table 1).

**Table 1:** E-cigarette flavors and their flavoring agents used to develop different flavored e-cigarette liquids.

Flavor	Flavoring agents	Reference
Tobacco	Ethyl maltol, 3-methylcyclopentane-1,2-dione, (E)-beta-damascone, dimethylhydroxy furanone, methylcyclopentanone, benzaldehyde, ethyl citrate, triacetin, racementhol, vanillin, ethyl lactate, ethyl vanillin, corylone, beta-Damascone, isoamyl butyrate	[13-16]
Piña colada	Allyl-cyclohexylpropionate, methyl 3-hydroxyhexanoate	[15]
Menthol	Menthol, limonene, carvone, pulegone, alpha-pinene, anethole, (E)-cinnamyl acetate, linalool	[14-16]
Mint	Menthol, benzoic acid, diacetate, benzaldehyde	[14]
Mango	Ethyl maltol, furanone, ethyl citrate, octadecanoic acid	[14]
Coffee	5-methyl-2-phenylhex-2-enal (Cocal), benzyl alcohol	[15,17]
Strawberry	Benzyl alcohol, gamma-decalactone, methyl cinnamate, 2,5-dimethylpyrazine, benzaldehyde, isovaleraldehyde, linalool	[15,18]
Cherry	Benzaldehyde	[19]
Fruit (such as fruit medley, passion fruit, orange, and guava)	Octadecanoic acid, furanone, menthol, benzoic acid, pyridine, cinnamic acid, maltol, limonene, furaneol	[14]
Cucumber	Hexadecanoic acid, benzoic acid, menthol	[14]
Crème brulee	Vanillin, benzaldehyde, benzoic acid, octadecenoic acid, squalene	[14]
Vanilla	Vanillin, ethyl vanillin, vanillin propylene glycol acetal, 2(3H)-furanone, para-anisaldehyde, benzaldehyde, ethyl maltol, pyridine, acetoin, dihydrojasmone, acetoin, sulfurol	[16,20]
Chocolate	2,5-dimethylpyrazine	[21]
Banana	Isoamyl acetate	[18]
Cinnamon	Cinnamaldehyde	[22]
Green apple	Farnesol, farnesene, hexyl acetate, ethyl acetate, and methylbutyl acetate	[23-25]
Grape	Ethyl acetate, ethyl butyrate	[17]
Clove	Eugenol	[18]
Burnt	Acetylpyridine	[18]
Butter	Diacetyl	[18]
Spicy	Eucalyptol	[18]
Cinnamon Apple	Vanillin propylene glycol acetal, furanone, coumarin, vanillin, ethyl vanillin, propenoic acid, maltol, ethyl maltol, cinnamaldehyde, pyridine, benzyl alcohol	[20]

What is known about the effect of flavoring agents on different organs?

As of 2021, at least 65 flavors in e-liquids or e-C aerosol were shown to illicit toxic responses in at least one organ system, including the respiratory tract and the cardiovascular system; the most frequently reported cytotoxic flavoring agent was cinnamaldehyde followed by vanillin, menthol, ethyl maltol, ethyl vanillin, benzaldehyde, and linalool [26]. Furthermore, these flavoring agents in e-C, including vanillin, ethyl vanillin (vanilla), and benzaldehyde (berry/fruit) can react with the e-liquid solvent, propylene glycol, to form acetals [27] that can efficiently transfer to e-C aerosol, and which may be more toxic compared with their parent aldehydes [28].

**Figure 1:**
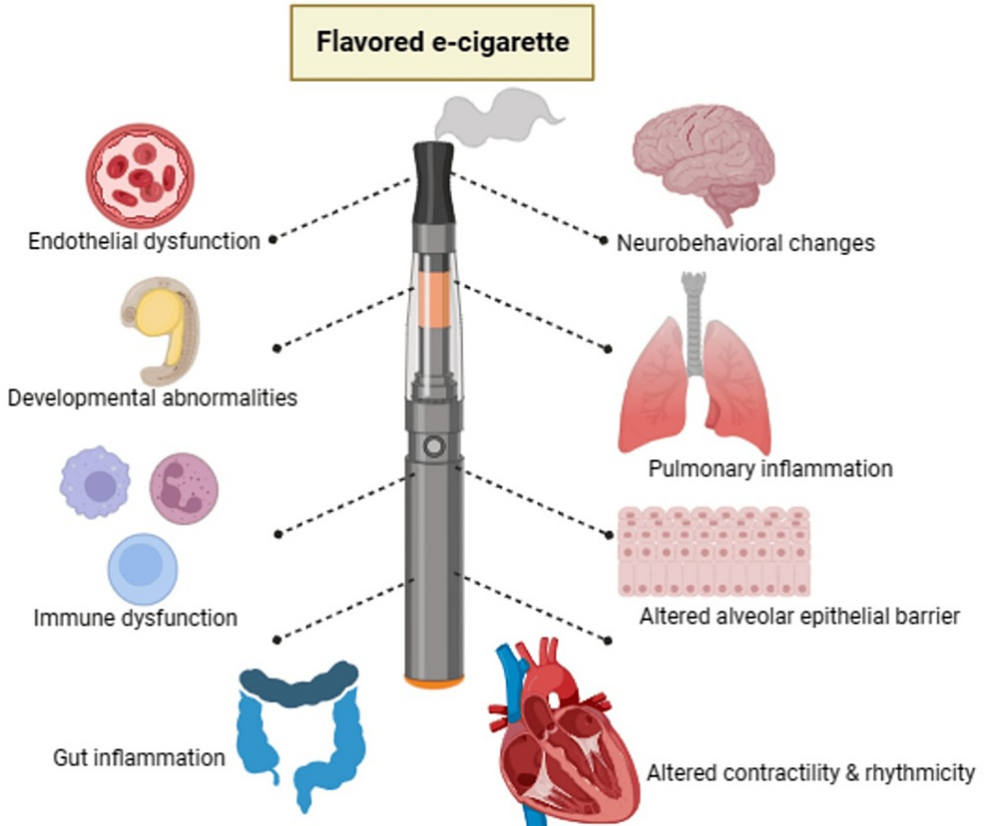
Effects of flavored e-cigarette aerosol or liquid in different organs based on in vitro and in vivo studies. The image was created by the authors of this study using BioRender.com.

The following deleterious effects represent the outcome of recent studies conducted on various organ systems exposed to flavored e-C aerosol or e-C liquid. Furthermore, both Figure 1 and Table 2 succinctly present the main finding(s) from these studies. Table 2 also contains information about animal, and/or human intact organs and organ-derived primary cells used for the study, duration of exposure, and flavoring agents or flavor (combination of flavoring agents) evaluated with or without nicotine.

**Table 2:** In vitro and in vivo studies showing the effects of flavors and their flavoring agents on different organ systems. e-C: electronic cigarette; e-liquid: electronic cigarette liquid; e-vapor: electronic cigarette vapor; ROS: reactive oxygen species; IL: interleukin; TEER: transepithelial electrical resistance; PGE2: prostaglandin E2α; LPS: lipopolysaccharide; RAGE: receptor of advanced glycation products; HMGB1: high mobility group box 1 protein; TNFα: tumor necrosis factor alpha; CXCL2: chemokine (C-X-C motif) ligand 2; COL1A1: collagen type I alpha 1 chain; MCP-1: monocyte chemoattractant protein-1; cGMP: cyclic guanosine monophosphate; VEGF: vascular endothelial growth factor; COX-2: cyclooxygenase-2; MUC5AC: mucin 5AC, oligomeric mucus/gel-forming gene; LC3: microtubule-associated protein light chain 3; NO: nitric oxide; NET: neutrophil extracellular trap; APD75: action potential duration at 75% repolarization; AP: action potential; CPP: conditioned plate preference; CPA: conditioned place aversion; pDA: putative dopamine neurons; pGABA: putative gamma-aminobutyric acid releasing neurons; nAChR: nicotinic acetylcholine receptors; VTA: ventral tegmental area; SNr: substantia nigra pars reticulata; TH+/DA: tyrosine hydroxylase dopamine neurons; TH-/non-DA: tyrosine hydroxylase non-dopamine neurons; hERG: human ether-a-go-go-related gene; I_Kr_: delayed rectifier potassium channel; DhβE: dihydro-β-erythroidine hydrobromide; ↑: increase(d), ↓: decrease(d); conc.: concentration; % v/v: volume per volume percent; WT: wild type; GFP: green florescent protein; eGFP: enhanced GFP; BAC: bacterial artificial chromosome

Variables	Study design	Flavor or flavoring agent used	Nicotine	Main findings	Reference
Endothelial dysfunction	Bronchial (BEAS-2B) and alveolar (A549) epithelial cells exposed to favoring agents and their propylene glycol (PG) acetals (0.3-10 mM) for 24 h	Benzaldehyde, vanillin, ethyl vanillin, and their corresponding PG acetals	Nicotine free	Benzaldehyde PG acetal and vanillin PG acetal showed conc. dependent ↓ in mitochondrial functions (oxygen consumption rate, ATP production, spare respiratory capacity, and maximal respiration). ↑ Cytotoxicity and ↓ cell proliferation	[28]
	Endothelial cells isolated by venous biopsy from non-smokers (n = 9, 5 female, 4 male, age 25-32 years), non-menthol (n = 6, 2 female, 4 male, age 30-50 years), and menthol cigarette smokers (n = 6, 2 female, 4 male, age 25-53 years) treated with 0.01 mmol/L menthol or eugenol; commercially available human aortic endothelial cells were treated with flavoring compounds (0.001-100 mmol/L) for 90 minutes	Vanillin, menthol, cinnamaldehyde, eugenol, dimethylpyrazine, diacetyl, isoamyl acetate, eucalyptol, and acetylpyrazine	Nicotine free	Impaired NO production was seen in endothelial cells from healthy participants. ↑ Cell death and ROS were induced only at high conc., and ↑ inflammation and NO at lower conc. of selected flavors (vanillin, menthol, cinnamaldehyde, eugenol, and acetylpyridine) in human aortic endothelial cells	[18]
	Human induced pluripotent stem cell-derived endothelial cells (iPSC-ECs) exposed to flavored e-liquids and 10% serum of e-C users (combined sole e-C users and dual e-C plus combustible cigarette users, average age 29 years) for 48 h	Fruit-flavored Rainier, sweet tobacco with caramel and vanilla-flavored RY4, tobacco-flavored Red Oak Tennessee Cured, sweet-flavored butterscotch, cinnamon-flavored Marcado, and menthol tobacco-flavored Tundra	0, 6, and 18 mg/mL	↑ ROS, caspase 3/7 activity, and low-density lipoprotein uptake, ↓ cell viability, impaired tube formation, and migration, ↑ IL-1β and IL-6 levels (macrophage polarization into a pro-inflammatory M1 state), ↑ ROS and impaired tube formation were seen in iPSC-ECs treated with serum from e-C users	[29]
	Porcine aortic endothelial cells were incubated with flavoring agent (1 mM each) for 10-75 min; isolated Sprague Dawley rat aortic rings precontracted with U-46619 were incubated with the flavoring compounds (0.1-1 mM each) for 30 min	Acetylpyridine, cinnamaldehyde, diacetyl, dimethylpyrazine, eucalyptol, eugenol, isoamyl acetate, menthol, and vanillin	Nicotine free	Cinnamaldehyde-inhibited Ca^2+^-stimulated endothelial cGMP accumulation, NO synthase activation, and soluble guanylate cyclase activity were seen in porcine aortic endothelial cells. Relaxation of pre-contracted aortic rings with flavoring compounds (most potent- eugenol and cinnamaldehyde) was not mediated by the endothelial NO synthase/cGMP pathway	[30]
	Bovine aortic endothelial cells exposed to 10% flavored e-C smoke extract (e-CSE) for 24 h; male C57BL/6 mice (10-12 weeks) skin wound treated with recombinant human VEGF + flavored e-CSE for 11 days	Classic tobacco, mint, menthol, vanilla/fruit from BLU or JUUL	0, 1.2%, and, 2.4% (BLU), and 3% (JUUL)	↑ Caspase 3 activity, endothelial superoxide, and ↓ NO bioavailability, and impaired monolayer wound closure were seen in bovine aortic endothelial cells. Impaired VEGF-dependent wound healing in diabetic mice after e-CSE treatment	[31]
Cardiac electrophysiological and functional alterations	Human induced pluripotent stem cell-derived cardiac myocytes (hiPSC-CM) treated with either (1-100 µM) parent cinnamaldehyde (no heating) or 100 µM heated cinnamaldehyde (prepared from low (200±50°C) and high (700±50°C) heat generated aerosol) solution for 6, 24, 48 h	Cinnamaldehyde	Nicotine free	Parent cinnamaldehyde induces time- and concentration-dependent ↓ in beating rate, impedance amplitude, beating frequency, and cell index. Longer exposure of parent cinnamaldehyde (≥20 min) at 100 µM conc. ↑ depolarization of resting membrane potential, loss of rhythmic action potential generation, and irregular patterns of AP triggering and repolarization. These changes were abolished with heated 100 mM cinnamaldehyde treatment.	[32]
	HL-1 mouse atrial cardiomyocytes and human induced pluripotent stem cell-derived cardiomyocyte (hiPSC-CM) treated with 0.075-0.75 puffs/mL flavored vapor extract for 24 and 48 h; C57BL/6 mice (5 months old, both sexes) were exposed to 4.7 s puffs of vanilla custard flavored e-vapor puff at 1.4 L/min, every 2 min for total of 60 puffs, 110 mL puff volume delivered over a 2 h period, 5 days a week, for a period of 10 weeks	Hawaiian passion fruit, orange, and guava (POG), vanilla custard (vanillin), and Apple Jax (cinnamaldehyde)	6 mg/mL	Dose-dependent ↓ HL-1 cell viability and ↑ apoptosis with vanilla custard and Apple Jax e-vapor. Both vanilla custard and apple jax e-vapor reduced beating rate and prolonged corrected field potential duration of hiPSC-CM via blockage of hERG encoded potassium current (I_Kr_). 10-week inhalational exposure to vanilla custard e-vapor resulted in ↓ parasympathetic activity seen by decreased pNN06 (percentage of adjacent NN intervals that differ from each other > 6 ms) and lower high-frequency component of heart rate variability, longer ventricular tachycardia episodes and prolonged APD75 at 15 Hz in e-vapor exposed mice.	[20]
	Female apolipoprotein E-deficient (ApoE^-/-^) mice were exposed to aerosols from 3 different e-vapor formulations as follows: carrier (propylene glycol and vegetable glycerol); base (carrier + nicotine); test (carrier + nicotine + flavor) and to cigarette smoke (CS) from 3R4F reference cigarettes for up to 6 months. The animals were exposed to 3R4F CS or e-vapor aerosols for 3 h/day, 5 days/week, in whole-body exposure chambers	Guaiacol (precursor of eugenol and vanillin)	4% nicotine	↑ Aortic arch area covered by atherosclerotic plaque formation was seen with CS but no such effect was found for any of the three e-vapor aerosols. ↑ Pulse wave velocity and arterial stiffness were significantly lower with base and test e-vapor exposure compared to CS. Ejection fraction, fractional shortening, cardiac output, and isovolumic contraction time remained unchanged following e-vapor exposure, but base and test e-vapor exposure caused an increase in isovolumic relaxation time (index of diastolic function) similar to CS	[33]
Inflammation	C57BL/6 female mice (6-8 weeks) exposed to flavored aerosol from JUUL pods in a 5-L whole-body exposure chamber for 20 min, 3 times/day, i.e., a total of 60 min/day for 1 or 3 months	Mango, mint	5% nicotine salts (59 mg/mL)	In the brain, ↑ inflammatory cytokines (TNFα, ILβ, IL6) were seen in the nucleus accumbens (NAc)-core or shell post 1 and 3 months exposure, significantly ↑ RAGE and its ligand HMGB1 were seen in the NAc-shell post 3-month exposure. In the cardiac tissue, ↓ TNFα, IL6, CXCL2, and COL1A1 with mint-flavor and ↓ CXCL2 with mango-flavor post 1-month exposure was observed but there was an increase in cytokines (TNFα, IL1β, IL6) and chemokines (CCL2, CCL3, CXCL1, CXCL2) with LPS treatment after 1 and 3-month mint-flavor exposure. In the colon, mango-flavor induced TNFα, IL6, and IL8 post-1 month exposure but levels ↓ post 3-month exposure compared to air or mint-flavor	[34]
	Human lung epithelial (NCI-H292) cell line exposed to flavored aerosol for 30 min. The puff regimen was 3 s puff duration, every 30 s, with a 55 mL puff volume for 30 min resulting in a total of 55 puffs	Tobacco, piña colada, menthol, coffee, strawberry	24 mg/mL	↓ Cell viability and metabolic activity and ↑ cytokine levels (IL1β, IL6, IL10, CXCL1, CXCL2, and CXCL10) showed differential effect with different flavors and devices with higher voltage. Coffee and strawberry were most toxic	[15]
	Human bronchial airway epithelial cells (H292) and human fetal lung fibroblasts (HFL1) were treated with 1-5% flavored e-liquid or 0.5-2.5% cigarette smoke extract (CSE) from 3R4F reference cigarettes with or without nicotine for 24 h; eight weeks old C57BL/6J mice were exposed to BLU e-C (classic tobacco flavored containing 16 mg nicotine) aerosol (200 mg/m^3^ TPM) for 5 h exposures per day for 3 successive days	Tobacco, cinnamon roll, grape vape, menthol, berry, melon, peach, pineapple, coconut, Mountain Dew, cotton candy, strawberry, marbo	0-24 mg	Fibroblast treated with either 1 or 5% tobacco-flavored e-liquid containing 0 or 24 mg nicotine or 1% CSE showed morphological alterations (enlarged cells with spindle formation and vacuolization) and decreased cell viability, which was exacerbated with nicotine. Cinnamon roll flavored e-liquid significantly ↑ IL-8 in fibroblast; tobacco flavor ↑ IL-6 and IL8 in the culture media of lung epithelial cells; ↑ MCP-1, IL-6, IL-1α, and IL-13 in the bronchoalveolar lavage of exposed mice; and reduced glutathione levels in the lung lysates of exposed mice	[35]
	Human bronchial epithelial cells (16-HBE and BEAS2B cell lines) and monocytes (U937 cell line) were exposed to aerosolized favors for 3 sessions of 30 min each at 12 h interval. For each session, a cell culture plate was placed inside the Enzyscreen chamber and the vapors were released (66 puffs during 22 min with a 3 s puff duration at 1.6 L/min flow rate and an inter-puff interval of approximately 17 s). The aerosols were allowed to equilibrate for 8 min post-exposure resulting in a total of 30 min per session before returning the cell culture plate to the incubator	Fruit Medley, Virginia tobacco, cool mint, crème brulee, cool cucumber, mango, classic menthol; other pod favors - just mango (strawberry coconut) and café latte	JUUL pod (5%) other pods (6%)	↑ Mitochondrial ROS, cell death, DNA fragmentation, IL-8, PGE2, IL-15, and other pro-inflammatory cytokines. ↓ TEER and voltage	[14]
	Human middle ear epithelial cells treated with flavored e-liquids (3.3% tobacco and 1.5% menthol) for 24 h	Tobacco and menthol	9.9 mg/mL	↓ Cell viability, ↑ inflammatory mediators COX-2 and TNFα levels, ↑ expression of MUC5AC (mucin production), and ↓ in epithelial sodium channels genes and ↑ aquaporin expression resulting in mucosal membrane expansion and fluid accumulation. ↑ Apoptotic marker, ↓ BCL-2, and ↑ autophagy marker LC3 were seen and more marked with menthol flavor	[36]
Developmental changes	Wild-type zebrafish embryos treated with cinnamon and chocolate-flavored e-vapor were added into dechlorinated water at 0.6, 12, and 25 puffs/L for 24 h	Cinnamon and chocolate	Nicotine free	Cinnamon-flavored e-vapor at 25 puffs/L significantly decreased end-diastolic and end-systolic volumes, stroke volume, heart rate, cardiac output, and red blood cell density except for blood vessel diameter	[37]
	Pregnant BALB/c mice (10 weeks) were exposed to cinnamon-flavored with nicotine e-C aerosols (3 s puff duration, and a 55 mL puff volume every 30 s) or HEPA-filtered air for 14-31 days, which included exposures for either 12 days before mating plus 19 days during gestation (preconception groups) or only during 14 days of gestation (prenatal groups). All preconception-exposed offspring were euthanized at birth and prenatal offspring were euthanized at birth or at 4 weeks of age	Cinnamaldehyde	36 mg/mL (nicotine salts)	↓ Body length and weight in offspring of both preconception and prenatally exposed dams. ↑ Tissue fraction in lung morphometric analysis, altered Notch 2 (epithelial cell differentiation), Shh and Wnt (lung organogenesis) pathways in offspring of both groups. Higher maximum respiratory elastance (elastic stiffness to expand) in preconception group dams. ↓ Maternal serum placental growth factor, increased 17β-estradiol and dysregulated maternal-fetal inflammatory genes (IL1β, IL4, IL6, STAT5A) in both groups	[38]
	Zebrafish embryos were exposed to e-C flavor dilution made in 1:1000 propylene glycol: embryo media from 6 hours post-fertilization (hpf) to 120 hpf	Bubble gum (ethyl acetate, ethyl butyrate, cinnamaldehyde), grape (ethyl acetate, ethyl butyrate), coffee, cotton candy, french vanilla, 555 (maltol, vanillin, ethyl vanillin), nicotine, unflavored	12 mg/mL and 24 mg/mL (present in nicotine flavor only), no nicotine in unflavored	Hyperactive behavior was seen in larval zebrafish at 120 hpf with all flavors, except for nicotine. Yolk sac and pericardial edemas, malformation of eye, jaw, cranial and cardiac regions, craniofacial deformity, bent body axis seen with all flavors at 120 hpf, most prominent effect seen with cinnamaldehyde containing flavor including increase in mortality at earlier time point, i.e., 24 hpf	[17]
	Fetal, neonatal, and adult ovine primary pulmonary artery smooth muscle cells (PASMC) were exposed to flavored e-liquid (1:100, 1:1000, and 1:10000) for 24 h; neonatal and adult ovine intrapulmonary arteries and the 5th generation bronchial rings treated with 1:1000 dilution of flavored e-liquid	Menthol, strawberry, tobacco, vanilla	Nicotine free	Menthol and strawberry flavors induced maximal cell death in both immature and adult PASMC with marked menthol toxicity in neonatal cells. Bronchodilation was more pronounced in the neonatal bronchial rings with menthol and tobacco flavors whereas adult rings only showed slight relaxation with menthol. Adult pulmonary artery relaxation was seen with menthol flavor	[16]
Epithelial barrier dysfunction	Human bronchial epithelial cells (16HBE14o) treated with flavored e-liquids (at conc. representing the % v/v present in e-C cartridges) for 24 h; Primary mouse tracheal epithelial (MTE) cells treated with 2,5-dimethylpyrazine (0.2% v/v present in e-C cartridge)	2,5-dimethylpyrazine (dark chocolate flavor), damascenone, linalool, α-ionone, ethyl maltol, furaneol and vanillin	Nicotine free	Loss of cell index (↓ in impedance, cytotoxicity) seen in flavored treated 16-HBE14o cells except with furaneol and vanillin due to solubility issues. 2,5-Dimethylprazine showed significantly reduced cell impedance in 16-HBE14o cells. ↑ Apical Cl^-^ ion conductance (short circuit current {I_sc_}) and ↓ transepithelial resistance in MTE cells	[21]
	Human lung epithelial and endothelial cells were exposed to flavored e-C condensed vapor for 24 h. The puff regime was set to 55 mL puff volume, 3 s puff duration, 60 s puff interval, and 200 puff (estimated maximum of puffs per day 235)	Cinnamon, tobacco, menthol	18 mg/mL	Cinnamon flavor ↑ cytotoxicity and pro-inflammatory response by ↑ IL-8 and MCP-1. ↓ TEER with both cinnamon and menthol-flavored aerosol	[22]
Immune dysfunction	Human alveolar macrophages (from bronchoalveolar lavage), neutrophils, and natural killer cells (from venous blood collected from healthy participants) were treated with flavored e-liquids (unheated) and vapored e-liquid condensate (VEC) (0.25, 0.5 and 1% dilutions) for 30 min. Vaped aerosol (1 puff of aerosol, every 30 s for 10 min resulting in 20 puffs total using an output setting of 60 watts) was allowed to cool and condense to generate VEC	Menthol (menthol tobacco and solid menthol), cinnamaldehyde (hot cinnamon candies, kola, and sinicide), isoamyl acetate (banana pudding and banana)	Nicotine free	Cinnamon-flavored e-liquids significantly ↓ phagocytic activity of both alveolar macrophages and neutrophils and suppressed NK cells' ability to eliminate target leukemia cells. ↑ IL-6 in macrophages, ↑ IL-8 production, and enhanced NET formation (anti-bacterial function) in neutrophils. Restoration of phagocytic activity of macrophages with small-molecule reducing agent 1,4-dithiothreitol. Cinnamaldehyde-flavored VEC did not significantly alter neutrophil phagocytosis compared to the unheated e-liquids	[39]
	Neutrophils isolated from venous blood of healthy human subjects were treated with 0-5 mM of flavoring agent for 1 h at 37°C	Cinnamaldehyde (cinnamon), ethyl vanillin (vanilla), benzaldehyde (almond or cherry), and isoamyl acetate (banana)	Nicotine free	Cinnamaldehyde and ethyl vanillin decreased total oxygen consumption in a dose-dependent manner. Cinnamaldehyde, ethyl vanillin, benzaldehyde, and benzaldehyde PG acetal significantly decreased neutrophil phagocytosis while isoamyl acetate had no effect	[40]
	Adult female C57BL/6 mice (8 weeks) were exposed to 70% vegetable glycerin and 30% propylene glycol (VG/PG) with or without French vanilla flavored e-C aerosol for 2 h/day, 7 days/week for 6 weeks in 5 L whole body exposure chamber. Vaping was conducted under a topography profile of 3 s puff duration, and a 55 mL puff volume every 30 s. E-C aerosols were sampled in the airstream exiting the chamber at a flow rate of 1 L/min throughout the experiment	French vanilla	Nicotine free	Greater tidal and minute volumes and ↑ lung tissue resistance were seen in vanilla-flavored exposed mice. Slight significant ↑ in NK cells and dendritic cell populations (CD11b+, CD11c+) exposed to flavored e-C aerosol. Increased T helper cell (CD4+) and B-cell (CD19+) populations in the lungs of mice treated with VG/PG regardless of flavor. Significant increase in IgG1 levels in the bronchoalveolar lavage fluid of mice exposed to VG/PG with vanilla-flavored e-C aerosol	[41]
Neurobehavioral effects	Eight-week-old male C57BL/6J mice were given drinking water containing nicotine (5 conc. - 30, 50, 75, 100, and 200 µg/mL) or nicotine-free (diluted to match the same fluid amounts) fruit- or tobacco-flavored e-liquids for one week in sequential order	Fruit or tobacco-flavored e-liquid (the chemical composition of fruit and tobacco flavoring in the e-liquids used was not mentioned in the present study)	18 mg/mL	Increased average daily consumption and proclivity for nicotine-containing fruit-flavored e-liquid but this increase was not due to flavor itself, average daily consumption of nicotine-free fruit and tobacco-flavored e-liquids increased compared to nicotine-containing flavored e-liquid and nicotine alone at the 200 µg/mL concentration. Total fluid intake (average total fluid consumption per week) and peripheral place conditioning assays (CPP and CPA) showed no difference with both flavored e-liquids with and without nicotine	[42]
	Adult C57/BL6J male mice (3-6 months) were used in (e-vape) vapor self-administration assays with e-liquids containing menthol ~15 mg/mL or green apple flavor 5-15 mg/mL with or without nicotine. Mice were placed in an air-tight chamber with 2 nose-pokes (one active and one inactive). Active nose-pokes resulted in a 3 s delivery of vaporized e-liquid with a 60 s timeout. A yellow cue light remained on during the timeout. For fixed-ratio 1 (FR1) schedule, mice began vapor self-administration on a Monday for 10 daily 3 h sessions for 5 days with weekend abstinence). Following FR1 escalation, mice transitioned to 3 h fixed-ratio 3 (FR3) sessions where they were maintained on their assigned e-liquid for 4 consecutive days to reach stable responding and re-baselined to their original FR1 assigned e-liquid on day 5. For progressive ratio (PR) schedule (after FR3 sessions and a weekend abstinence), the following equation was used (PR = 2^(2n/9)^, where “n” is the number of earned e-vape deliveries within the session) to determine the number of active nose-pokes required for e-vape delivery for each 3 h session for 4 days and then re-baselined for 2 days. During PR sessions, the active and inactive nose-poke was set to match FR1 and FR3 sessions for the first 2 days and then reversed on the next 2 days to examine presence of side bias; DhβE, an α4β2 nAChR antagonist, was intraperitoneally injected (2 mg/kg) prior to self-administration sessions (on day 1 and 2 with saline, day 3 and 4 with DhβE) when used	Menthol and green apple	6 mg/mL	Menthol + nicotine and green apple alone produced the highest number of FR1 e-vape deliveries followed by green apple + nicotine and nicotine alone on FR1 escalation schedule. Nicotine, green apple, green apple + nicotine, and menthol + nicotine produced significantly more FR3 deliveries, marked effect seen with both flavors plus nicotine compared to nicotine alone. DhβE significantly reduced FR3 responses in mice assigned to nicotine, green apple + nicotine, and menthol + nicotine. PR sessions showed no side bias in self-administration behavior, similar trend was observed in both PR and FR3 e-vape deliveries	[25]
	Adult male and female C57BL/6J mice (3 - 6 months) genetically modified to contain fluorescent nicotinic acetylcholine receptors (nAChRs) (α4-mCherryα6-GFP mice) were implanted with menthol osmotic pumps (2 mg/kg/h) with or without nicotine (2 mg/kg/h) for 10 days (for in vivo upregulation assays and chronic treatment). For CPP assay, non-transgenic (WT) littermates of α6-GFP BAC transgenic adult mice were administered intraperitoneal injection of 1 mg/kg menthol with or without 0.5 mg/kg nicotine on alternating days for a total of 8 days. For electrophysiological assay cultured midbrain neurons from tyrosine hydroxylase (TH)-eGFP mice were treated with 500 nM menthol with or without 200 nM nicotine for 10 days. For all experiments, 3-6 months adult mice were used; mouse neuroblastoma 2a (neuro-2a) cells were transiently transfected with α4-GFPβ2 or α6-GFPβ2β3 nAChR subunits and treated with nicotine (50 or 100 nM) or menthol (500 nM) plus nicotine for 24 h	Menthol	0.5 mg/kg	↑ CPP (reward-related behavior) in nicotine + menthol and nicotine alone treated mice. ↓ Baseline firing frequency in TH+/DA and ↑ in TH-/non-DA (pGABA) neurons after menthol (500 nM) + nicotine (200 nM) treatment was more than nicotine alone. TH+/DA neurons treated with menthol plus nicotine exhibited an ~ 8-fold increase in firing frequency following nAChR activation. Menthol enhances only α4 containing nAChR upregulation in VTA DA and SNr GABA neurons and does not enhance α6 containing nAChR upregulation. In the transfected neuro-2a cells, menthol alone increases the number of low-sensitivity α4_(3)_β2_(2)_ nAChRs and low-sensitivity α6β2 (non-β3) nAChRs	[43]
	Adult male and female C57BL/6J mice (3-6 months) genetically modified to contain fluorescent nicotinic acetylcholine receptors (nAChRs) (α4-mCherryα6-GFP mice) were administered intraperitoneal injection of farnesol (0.1-10 mg/kg) with or without nicotine (0.5 mg/kg); mouse neuroblastoma 2a (neuro-2a) cells transfected with α4-GFPβ2 and β2WT nAChR subunits and treated with 500 nM farnesol for 24 h	Farnesol (green apple)	0.5 mg/kg	Reward-related behavior in CPP assay was observed with 1 mg/kg farnesol-alone and additive effect with nicotine in male mice only. ↑ Locomotor activity in both sexes. ↑ Firing frequency of pDA due to ↓ pGABA firing frequency in farnesol (1 mg/kg) treated neurons from male mice due to upregulation of α6 and α4α6 containing nAChR on VTA pDA neurons and ↓ in α4 containing nAChR in SNr GABA neurons. Farnesol + nicotine has no additive or enhancing effect on nAChR upregulation, it counteracts both the effect of farnesol alone to upregulate α6 containing nAChR on VTA pDA neurons and the effect of nicotine alone on α4 containing nAChR upregulation on SNr GABA neurons. In the transfected neuro-2a cells, farnesol showed concentration-dependent (0-300 µM) inhibition of α4β2 nAChR function and long-term farnesol treatment (500 nM, 24 h) facilitated α4β2 nAChR recovery following desensitization	[23]
	Adult male and female C57BL/6J mice (3-6 months) genetically modified to contain fluorescent nicotinic acetylcholine receptors (nAChRs) (α4-mCherryα6-GFP mice) were administered intraperitoneal injection of farnesene (0.1-10 mg/kg) with or without nicotine (0.5 mg/kg); mouse neuroblastoma 2a (neuro-2a) cells transfected with α4-mCherry, α4-GFP, and β2WT or α4-mCherry, α6-GFP, and β2WT) nAChR subunits and treated with 500 nM farnesene for 24 h to examine changes in α4α6β2 and α4β2 nAChR stoichiometry	Farnesene (green apple)	0.5 mg/kg	Lowest dose of farnesene (0.1 mg/kg) produced a significant rewarding effect in CPP assay in both sexes, higher doses showed no change from baseline in male mice and female mice exhibited a significant change from baseline with all farnesene doses when compared with saline. No significant change in locomotory activity with farnesene in both male and female mice. Both sexes exhibited enhanced rewarding effects in the nicotine + farnesene group compared with nicotine alone, but only females exhibited a significant enhancement. Farnesene treatment produced no significant changes in nAChR number within pDA or GABA neurons in the VTA or SNr. Farnesene enhances nicotine’s potency for activating nAChRs on VTA dopamine neurons by altering nAChR stoichiometry on putative VTA dopamine neurons toward high-sensitivity α4β2 nAChRs. Farnesene treatment promotes high-sensitivity α4_(2)_β2_(3)_ nAChRs by decreasing the number of low-sensitivity α4_(3)_β2_(2)_ and α4α6β2 nAChRs in neuro-2a transfected cells	[24]

Endothelial dysfunction

Studies have shown that flavoring agents alter vascular endothelial cell function. In a study conducted by Fetterman et al., researchers showed that endothelial cells isolated from non-smokers (n = 9; 5 female and 4 male aged 24-32 years old) when treated with either menthol or eugenol (0.01 mmol/L) resulted in decreased nitric oxide (NO) production. They also demonstrated the concentration (0-100 mmol/L) dependent effect of e-C flavoring agents on a commercially available human aortic endothelial cell line. Cell death and ROS production were observed only when cells were incubated for 90 min at very high concentrations (10-100 mmol/L) with certain flavoring agents, such as vanillin, menthol, cinnamaldehyde, eugenol, dimethylpyrazine, diacetyl, isoamyl acetate, eucalyptol, and acetylpyrazine. However, lower concentrations (0.01-10 mmol/L) of some flavoring agents (vanillin, menthol, cinnamaldehyde, eugenol, and acetylpyridine), on the other hand, induced inflammation and impaired NO production suggesting endothelial dysfunction [18]. Another study conducted in human-induced pluripotent stem cell-derived endothelial cells (iPSC-ECs) from healthy individuals treated with flavored e-liquids with varying nicotine concentrations showed variability in cytotoxic effects for different flavored e-liquids. Cinnamon and menthol-flavored e-liquids were highly potent and significantly decreased cell viability, increased reactive oxygen species, apoptotic signals (caspase 3 and caspase 7 activity), activation of oxidative stress pathways, low-density lipoprotein (LDL) uptake and inhibited endothelial cell tube formation, and migration. They also showed that macrophages exposed to conditioned media obtained from iPSC-ECs treated with both cinnamon and menthol-flavored e-liquids with nicotine (18 mg/mL) expressed both CD40 (M1 pro-inflammatory marker) and CD163 (M2 anti-inflammatory marker) and increased production of related inflammatory cytokines, such as interleukin (IL)-1β, IL-6 and IL-10 mainly with cinnamon treatment. In the same study, treatment of iPSC-ECs with serum collected from sole e-C users and dual users (both e-C + combustible cigarette) of average aged 29 years and younger resulted in increased reactive oxygen species (ROS) production and impaired endothelial tube formation [29].

A study in precontracted aortic rings showed that flavoring agents (eugenol, cinnamaldehyde, vanillin, menthol, acetylpyridine, and 2,5-dimethylpyrazine) induced vascular relaxation with EC50 values ranging from 0.5 mM to 4.2 mM. Surprisingly, vasorelaxation seen with all the flavoring agents was not inhibited by nitric oxide synthase (NOS) inhibitor, N(gamma)-nitro-L-arginine methyl ester (L-NAME) suggesting a lack of direct involvement of endothelial eNOS/cyclic guanosine monophosphate (cGMP) pathway unlike some of the concurrent studies. However, both eugenol and cinnamaldehyde inhibited calcium chloride (CaCl_2_) induced contraction in rat aortas and possibly produced maximal relaxation by altering calcium flux in the vascular smooth muscles [30].

Studies on flavored e-C (classic tobacco, mint, menthol, vanilla, or fruit) smoke extract with varying nicotine concentration (1.2-3%) on bovine aortic endothelial cells demonstrated endothelial dysfunction by increased superoxide production, caspase 3 activity, and decreased NO bioavailability in a nicotine content dependent manner. In addition, impaired angiogenesis and wound healing were evident in both endothelial cells and diabetic mice treated with e-C smoke extract with varying nicotine content. Endothelial cell monolayer wound closure failed due to reduced proliferation and migration of cells and wound healing ability of angiogenic protein; vascular endothelial growth factor was also inhibited in diabetic mice [31].

Taken together, these studies suggested that exposure to flavored e-liquids even at subclinical concentrations can increase oxidative stress, induce inflammatory response, decrease NO bioavailability, and impair angiogenesis resulting in endothelial dysfunction.

Cardiac electrophysiological and functional alterations

Few studies have revealed that flavoring agent exposure decreases cell viability and alters electrophysiological function of cardiomyocytes. Nystoriak et al. in 2019 tested the cardiac toxicity of parent or heated cinnamaldehyde on electrical signaling activity of human induced pluripotent stem cell (hiPSC)-derived cardiomyocytes after acute or prolonged exposure. They found a concentration-dependent reduction in cell index (measure of electrical impedance; reduction relative to baseline indicates cell death), impedance amplitude (number of positive and negative cell index peaks), beating frequency (number of positive and negative peaks in 20s) and beating rate, and nearly 30% reduction in cell viability after 24-48 h exposure with 100 µM parent cinnamaldehyde. Prolonged application for about 20 minutes of 100 µM concentration of parent cinnamaldehyde caused progressive depolarization and loss of rhythmic action potential spiking activity [32].

Abouassali et al. in 2021 showed that vanillin and cinnamaldehyde were more toxic to HL-1 cardiomyocytes than fruit-flavored e-vapor. In spontaneously beating hiPSC-derived cardiomyocytes, cinnamaldehyde (Apple Jax flavor) or vanillin (vanilla custard flavor) flavored e-vapor affected the beating rate (BR) and prolonged the field potential duration (FPD) of these cells to a greater extent than fruit-flavored e-vapor (Hawaiian passion fruit, orange, and guava {POG} flavor), which was accentuated with nicotine [20]. They found that vanillin-flavored e-vapor significantly reduced repolarizing human ether-a-go-go-related gene (hERG) encoded potassium current (delayed rectifier potassium channel, I_Kr_), known to regulate electrophysiological parameters, in response to -60 to +60 mV in HEK293 cells transfected with hERG ion channel. In the same study, when mice were exposed to vanillin aldehyde-flavored e-vapor for 10 weeks, an increased sympathetic predominance was observed. Decreased temporal heart rate variability (HRV) variable, percentage of consecutive NN intervals that differ from each other by >6 ms (pNN06), no change in low frequency (LF) component but significant reduction in high frequency (HF) component of HRV spectrum (marker of cardiac vagal tone) indicated reduced parasympathetic activity. In addition, inducible ventricular tachycardia was of a longer magnitude, and ventricular action potential duration alternans was longer compared to control mice. Thus, the authors confirmed that electronic nicotine delivery devices that included these flavors resulted in action potential instability and inducible ventricular arrhythmias [20]. In a study by Szostak et al. in 2020, e-vapor aerosol-containing flavor exposure to apolipoprotein E-deficient mice did not accelerate atherosclerosis, while exposure to standard cigarette smoke did worsen this process. E-vapor aerosol containing flavor (guaiacol or 2-methoxyphenol is a precursor of eugenol and vanillin) and nicotine did not alter left ventricular fractional shortening or ejection fraction, but it increased isovolumic relaxation time of the left ventricle, suggesting a diastolic abnormality [33].

These studies highlighted that prolonged exposure to these flavoring agents decreases cell viability and alters the electrophysiological function of cardiomyocytes, possibly by blocking hERG current (I_Kr_), and could lead to tachycardia and arrhythmogenesis. Among the various commercially available flavoring agents, cinnamaldehyde and vanillin produced a more pronounced impairment of cardiomyocyte rhythmicity and contractility of cardiomyocytes.

Inflammation

In an in vivo study recently reported by Moshensky et al. in 2022, authors investigated the effect of inhalation of aerosols from flavored (mint, mango) pod-based (JUUL) e-C containing nicotine salts (59 mg/mL) for 4-12 weeks on markers of inflammation in multiple organs. JUUL aerosol exposure increased inflammatory genes and cytokines in the brain and colon. They demonstrated that mint and mango flavored e-C aerosols upregulated expression of inflammatory cytokines, TNF-α, IL-1β, IL-6, and protein levels of receptors for advanced glycation end products (RAGE) and its ligand high mobility group box 1 (HMGB1) in the nucleus accumbens-shell region of the brain after four and 12 weeks of aerosol exposure. The nucleus accumbens serves as a key motor-limbic interface regulating reward and pleasure processing behavior and increased expression of neuroinflammatory marker, HMGB1 in this region of the brain could be associated with addiction observed in vapers. In the colon, mango-flavored aerosol induced inflammation by upregulation of tumor necrosis factor (TNF)-α, IL-6, and IL-8 after four weeks of exposure. To the surprise of the investigators, exposure to the e-C aerosols did not upregulate pro-inflammatory cytokines or chemokines in the heart. In fact, some were downregulated. However, in another series of experiments, in which acute lung injury was induced with lipopolysaccharide, the cardiac inflammatory response was exacerbated by e-C aerosol. Interestingly, no pathological changes were apparent at cellular (leukocyte and neutrophil count in bronchoalveolar lavage), histological (increased airspaces and inflammatory cell infiltration), and functional (emphysema commonly seen in smokers) levels in the lungs, and no alteration in functional parameters (blood pressure, heart rate, and heart rate variability) seen in the heart of mice exposed to both flavors for 4-12 weeks. However, RNAseq identified significant changes in the expression of 155 genes with mango flavor and 74 with mint flavor which included GTPases (nicotine), mucins (mint flavor), chemokine (C-C motif) ligand 6 (CCL6) (mango flavor), and transforming growth factor-β (TGFβ) receptors (nicotine and mango flavor). These alterations could induce harmful effects in the long term, particularly with mint flavor which showed increased changes with chronic 12 weeks exposure [34].

In a 2016 study, Leigh et al. showed that different ENDS reduced cell viability and metabolic activity of H292 human bronchial epithelial cells either equivalent to or to a lesser extent compared to the reference tobacco cigarettes. The inflammatory cytokines were significantly upregulated although their levels differed with different ENDS products. Notably, out of three flavors (menthol, coffee, and strawberry) delivered by tank-type ENDS that significantly increased cytotoxicity, inflammatory response, and decreased metabolic activity, strawberry was found to be the most toxic [15].

It was also demonstrated that oxidant reactivity of both unvaporized and vaporized ENDS e-liquids was enhanced by the addition of flavors with sweet or fruit flavors being stronger oxidizers [35]. Both human airway epithelial cells and fibroblasts showed increased levels of inflammatory cytokines; fibroblasts also exhibited phenotypic alterations, such as enlargement, vacuolization and spindle formation, loss of fusiform structure, and reduced cell viability. As compared to tobacco and grape flavors, cinnamon-flavored e-liquid elicited higher inflammatory response by upregulating cytokine and IL-8 levels in the lung fibroblasts. Human bronchial airway epithelial cells (H292) when directly exposed to tobacco-flavored aerosol for 10 min using air-liquid interface culture system demonstrated a significant increase in cytokine IL-6 and IL-8 in culture media measured 16 h post exposure. In the same study, bronchoalveolar lavage of mice treated with tobacco-flavored e-C aerosol showed increased cytokines, such as monocyte chemoattractant protein (MCP)-1, IL-6, and decreased glutathione levels in the lung lysates.

Another study by Muthumalage et al. in 2019 showed that all the tested e-cigarette flavored pods (seven flavors with 5% nicotine by JUUL, one with unlisted nicotine concentration by LCF labs, and one with 6% nicotine by Eon Smoke labs) dose-dependently increased acellular reactive oxygen species (ROS) production, but JUUL flavor classic menthol induced more pronounced generation of mitochondrial ROS in the lung epithelial cells. These flavored pods reduced transepithelial voltage and resistance, increased DNA damage in monocytes, and increased pro-inflammatory cytokines, IL-8, prostaglandin E2 (PGE2), and TNFα in both lung epithelial cells and monocytes. Among the tested flavors, which differed in their activation of different cytokines and chemokines, the strawberry coconut flavor (sold as “Just Mango” by LCF labs) was the most potent inducer of TNFα in epithelial cells [14].

A few researchers have also investigated the extra cardiopulmonary effects of flavoring agents on cells isolated from other tissues. A study conducted on human middle ear epithelial cells (HMEEC) showed that out of 73 flavored e-liquids from 12 commercial brands, menthol, and tobacco-flavored e-liquids were the most toxic flavors. Both flavored e-liquids decreased cell viability of HMEEC by upregulating inflammatory cytokines, cyclooxygenase-2 (COX-2), and TNFα, activating apoptosis and autophagy programmed cell death, and dysregulation of epithelial sodium or aquaporin water channels [36].

In addition to cytotoxicity and exacerbation of inflammatory response, these studies have revealed that flavoring agents have the potential to modulate reward-related behavior by upregulating the neuroinflammatory marker, HMGB1 in the nucleus accumbens. They also increased ROS generation, reduced antioxidant glutathione and metabolic activity of lung epithelial cells, induced phenotypic changes in fibroblast, DNA damage in monocytes, and dysregulation of epithelial sodium and aquaporin water channels.

Developmental effects

There is some in-vivo evidence that suggests cinnamon-flavored, nicotine-free, e-C vapor can impede cardiovascular function during early development. A study on zebrafish embryos by Piechowski and Bagatto in 2021 showed that a high dose of nicotine-free cinnamon-flavored e-C exposure but not chocolate-flavored e-C vapor depressed all cardiac parameters (end-systolic volume, end-diastolic volume, stroke volume, heart rate, and cardiac output) except for blood vessel diameters. The authors concluded that cinnamon-flavored e-C vapor can depress cardiovascular function during the early development of the heart and in the absence of nicotine [37].

Noël et al. in 2020 demonstrated that nicotine-containing cinnamon flavored e-cig with nicotine (36 mg/mL) aerosol exposure during preconception and prenatal period resulted in altered maternal-fetal lung immune-inflammatory response, decreased maternal serum placental growth factor and increased maternal serum 17β-estradiol indicating disrupted maternal endocrine environment and reduced birth weight, which remained low through four weeks of age in prenatally exposed offspring, and smaller birth length of the offspring. Lung morphology also showed a significant decrease in parenchymal airspace at birth in the offspring of both preconception and prenatal exposed dams indicating impaired lung organogenesis. The molecular analysis elicited dysregulation of the signaling pathways, Notch 2, Shh, and Wnt that are implicated in the airway epithelial cell differentiation, growth, and maturation [38]. 

In another study, Holden et al. showed that zebrafish embryos were exposed to non-combusted eight e-cigarette flavored liquids containing benzyl alcohol, cinnamaldehyde, ethyl butyrate, vanillin, ethyl vanillin, maltol, and ethyl acetate from 6 h post fertilization (hpf) to 120 hpf. The results revealed morphological changes in the yolk sac, pericardial edema, eye abnormality, bent body axis, and jaw deformities observed at 120 hpf. The study also identified the specific flavoring agents associated with morphological alterations. The highest incidence of cranial and cardiac malformations was seen with flavors containing ethyl vanillin, maltol, and vanillin. Notably, the two flavors bubble gum and grape differed in their morphological toxicities despite sharing similar composition, both contained ethyl acetate and ethyl butyrate. Conceivably, the presence of cinnamaldehyde in bubble gum flavor resulted in a four-fold increase in percent mortality compared to grape flavor making bubble gum the sole favor associated with highest mortality at 24 hpf. Furthermore, at 120 hpf, there was a notable hyperactivity in larval photomotor response (LPR) to all flavors except nicotine flavor. Nicotine flavor exposure exhibited hypoactivity in LPR which could be due to overstimulation of nicotinic acetylcholine receptors [17].

In the 2019 study, Berkelhamer et al. made noteworthy observations regarding flavored nicotine-free e-cigarette solutions. They found that even at lower concentrations, particularly in the case of menthol and strawberry flavors, these solutions triggered significant cell death in both mature and immature pulmonary artery smooth muscle cells. Interestingly, the toxicity of menthol was more pronounced in neonatal cells. When exposed to various nicotine-free flavored solutions, neonatal precontracted bronchial rings exhibited bronchodilation, more marked effects seen with menthol and tobacco-flavored solutions. In contrast, among all the flavors tested, only menthol demonstrated the ability to dilate precontracted adult bronchial rings and pulmonary arteries. It's worth noting that none of the flavors had any discernible impact on the vascular response of neonatal pulmonary arteries [16].

These studies suggest that flavored e-cigarette vapor with or without nicotine could adversely affect organogenesis, weaken maternal-fetal immune function, induce both morphological and functional birth defects in different organs, particularly in brain, heart, and lung, cell death of neonatal and fetal pulmonary artery smooth muscle cells and decreased bronchial and pulmonary arteries vasoreactivity.

Epithelial barrier dysfunction

Although there is a deluge of studies assessing the effect of e-C on lung epithelial cells viability and inflammation, only a few studies were focused on the effect of flavoring agents, on epithelial barrier functional alterations.

Sherwood and Boitano in 2016 highlighted that primary mouse tracheal epithelial cells treated with 2,5-dimethylpyrazine (dark chocolate flavor) showed an increase in apical airway surface Cl^-^ ion efflux (measured as an increase in short-circuit current {Isc}) and transient loss of transepithelial resistance. Mechanistic experiments revealed that the increase in apical airway surface ion (Cl-) conductance was due to cyclic adenosine monophosphate-protein kinase A cascade (cAMP/PKA) dependent activation of the cystic fibrosis transmembrane conductance regulator (CFTR) ion channel. In the same study, human bronchial epithelial cells (16HBE14o) when subjected to different flavoring agents at concentrations similar to those found in commercial e-C liquid for 24 h, displayed a decrease in cell index. This reduction in cell index, which is determined by measuring relative cellular impedance measured before and after the addition of ATP, serves as an indicator of cytotoxicity. Human bronchial epithelial cells exposed to sub-cytotoxic concentrations of 2,5-dimethylpyrazine for 24 h showed significantly reduced ability to elicit physiological response (e.g., mucociliary clearance) to both forskolin and exogenous ATP (to a lesser extent), which are important signaling molecules involved in airway innate immunity [21].

Bengalli et al. in 2017 demonstrated that menthol and cinnamon-flavored condensed aerosols synergistically reduced transepithelial electrical resistance (TEER), induced cytotoxicity with nicotine in human epithelial alveolar cells, and altered alveolar-blood barrier integrity. An increase in pro-inflammatory cytokines IL-8 and MCP-1 was observed with cinnamon-flavored condensed aerosols only [22].

Taken together, these studies revealed that cinnamon and dark chocolate flavors were found to induce cytotoxicity and disrupt the integrity of the alveolar blood barrier. This disruption led to an increase in ion conductance, primarily attributable to the activation of the cAMP/PKA/CFTR signaling cascade, ultimately resulting in a loss of transepithelial electrical resistance.

Immune dysfunction

In another study, Clapp et al. in 2017 showed that seven flavored nicotine-free e-liquids containing menthol, cinnamaldehyde and isoamyl acetate abrogated cell-mediated cytotoxic response in natural killer (NK) cells, impaired neutrophil extracellular trap formation characterized by nuclear membrane disintegration, chromatin extrusion and loss of nuclear and granular integrity, and increased IL-6, IL8 production in neutrophils and alveolar macrophages isolated from healthy participants. Immune cell dysfunction was seen with reduced phagocytic capacity of macrophages and neutrophils and NK cell killing of target leukemia cells was more prominent in 3 cinnamaldehyde-containing flavored e-liquids. However, treatment with thiol-reducing agent 1,4-dithiothreitol (DTT) reversed the inhibiting effect on phagocytic ability of macrophages, possibly by altering the sulfhydryl-modifying activity of cinnamaldehyde present in cysteinyl groups of proteins [39]. In addition, Hickman et al. in 2019 also showed that flavoring agents such as cinnamaldehyde, ethyl vanillin, benzaldehyde, and benzaldehyde propylene glycol present in e-liquids dose-dependently decreased intrinsic neutrophil function seen as decrease in total oxygen consumption during oxidative burst (i.e., amount of oxygen converted into superoxide) and impaired their phagocytotic ability. The authors proposed that decreased oxidative burst could be due to reduced glucose uptake, change in nicotinamide adenine dinucleotide phosphate (NADPH) oxidase function, or dysregulation of the metabolic pathways [40].

A study by Szafran et al. in 2020 showed that mice exposed to nicotine-free vanilla-flavored e-liquid had an increase in lung tidal and minute volumes. There was an increase in lung tissue resistance, which could be attributed to changes in peripheral lung function caused by increased infiltration of the lung tissue with NK cells, dendritic cells (CD11b+, CD11c+) with vanilla flavor, and T-helper cells (CD4+) and B cells (CD19+) exposed to e-C aerosol irrespective of vanilla flavor [41].

These findings suggest that flavoring agents increased infiltration of immune cells but impaired phagocytotic function of NK cells and neutrophils, decreased intrinsic neutrophil function, and ability to form neutrophil extracellular trap formations.

Neurobehavioral effects

There are a few animal and human studies that have shown that flavoring agents can induce addictive behavior and enhance nicotine-associated dependence. Wong et al. in 2020 demonstrated that mice voluntarily consumed fruit-flavored nicotine e-liquid more than nicotine alone or nicotine-free fruit-flavored e-liquids. This preferential behavior indicates a tendency for higher use of e-cig with fruit flavors and consequently, increased nicotine intake and addiction [42]. In a similar study, Cooper et al. in 2022 showed that male mice exhibited escalation of e-vapor self-administration behavior with both menthol and green apple flavor with nicotine and green apple alone attributed to β2-containing (β2*) nicotinic acetylcholine receptors (nAChR)-mediated mechanism [25]. Investigators also conducted studies to evaluate menthol-induced enhancement of nicotine reward reinforcement behavior. They found that menthol treatment increases number and nicotine-induced activation of nicotinic acetylcholine receptors expressed on midbrain dopaminergic (DA) neurons, which are involved in reward-related behavior. Following nAchR activation, firing frequency was increased eight-fold after menthol plus nicotine 10 days treatment of midbrain cultured DA neurons isolated from tyrosine hydroxylase (TH+) expressing green fluorescent protein (eGFP) mice. Microscopy assays revealed that menthol plus nicotine upregulated nAchR subtypes, α4-containing (α4*) and α4α6-containing (α4α6*) nAChRs in ventral tegmental area dopaminergic (VTA DA) and substantia nigra pars reticulata (SNr) GABAergic neurons, increased DA neuron spontaneous firing and enhanced nicotine reward-related behavior in GFP mice [43]. The same research group also showed that farnesol and farnesene, present in green apple and fruit flavors showed differential effects on both locomotor activity and reward-related behavior. Farnesol increased ambulatory behavior in both sexes whereas farnesene showed no change. A lower dose (0.1 mg/kg) of farnesene elicited reward-related behavior in both male and female mice but structurally similar farnesol-produced effect in male mice only. Furthermore, farnesol acts by stimulating VTA DA firing frequency possibly by decreasing GABAnergic inhibitory neuronal function [23]. On the other hand, farnesene alters nAchR stoichiometry by upregulating high sensitivity α4β2 nAchR in VTA DA neurons [24]. The authors confirmed that flavoring agents activate nicotinic acetylcholine receptors, which could exert addictive reward & reinforcement behavior beyond their flavoring and odorant effects in a sex-dependent manner depending on the flavoring agent used.

Pullicin et al. in 2020 demonstrated that the addition of cherry flavor affected the sensory perception and appeal to e-C aerosol. Flavored e-C aerosol increased perceived sweetness and improved liking but increasing concentration of nicotine nullified the pleasing effect of the sweet flavor [44]. Further, it was also shown that e-C smokers preferred their usual brand but demonstrated higher average puff duration with strawberry flavor as compared to tobacco [45]. Vaping topography and use patterns assessed by puff duration, inter-puff interval, and number of puffs taken were associated with increased nicotine intake and exposure to usual brands of e-C users. However, it varied with the tested flavors possibly due to different concentrations of nicotine.

In summary, both animal and human studies showed that flavoring agents enhance the reward-related behavior of nicotine and increase e-C usage possibly, by activating nAChR resulting in increased dopaminergic neuronal firing.

Limitations

The studies described above demonstrate several strengths, including their assessment of flavorings directly on endothelial and epithelial barriers and immune cells, utilizing an in vitro approach that allows for high throughput screening of various flavors. However, these studies also have limitations. Few studies have comprehensively assessed the effects of multiple flavorings on both in vitro and in vivo long-term outcomes. With hundreds of flavoring agents available, it is challenging to study the effects of each agent on different organ systems. Most studies have focused on popular flavors rather than individual flavoring agents. Moreover, there has been a predominance of in vitro models used, with limited animal models or clinical studies conducted. Comprehensive evaluation of the long-term effects of flavors on in vivo cardiovascular physiology, including electrical activity, heart rate variability, left ventricular function, vascular function, platelet aggregability, and cardiac gene expression, lung injury, neurobehavioral disorders, including cognitive impairment, and other organ-specific abnormalities remain scarce.

The studies included in this review have primarily examined the effects of flavors with or without nicotine, making it challenging to determine the specific role of flavors alone in causing deleterious effects, as they may interact with nicotine and other constituents of e-cigarette aerosols. Although our search on PubMed focused on studies investigating the effects of flavors used in e-cigarettes on different organs, there is a scarcity of research explicitly dedicated to flavors or their chemical constituents, flavoring agents. Therefore, we have compiled a limited number of in vitro and in vivo studies available in the literature. It is important to note that this is not a meta-analysis, and further research is necessary, including investigations in both animal models and humans, to gain a clear understanding of the effects of flavors. Additionally, the limited number of human studies conducted on flavors in e-cigarette use does not provide sufficient evidence to claim their role in addiction. However, based on the available studies, it has been observed that flavors or flavoring agents produce a pleasurable effect, and individuals report higher satisfaction with vaping.

Future direction

Despite numerous investigations, there exists a gap in knowledge regarding the long-term impact of flavor additives in e-cigarette aerosols on cardiopulmonary, vascular, and neurological functions. In vitro studies suggest that certain flavors may exert direct toxic effects on endothelial and cardiac cells, but whether chronic exposure to these flavors leads to sustained cardiac pathology and physiological abnormalities in vivo necessitates further investigation. Additionally, it remains unclear whether chronic exposure to different flavoring agents, in combination with nicotine in e-cigarette vapor, alters the aerosol type and chemical composition compared to nicotine alone. The formation of adducts resulting from nicotine and flavoring interactions could potentially exacerbate cardiovascular and neurological pathophysiology. Furthermore, there is a lack of clinical studies that specifically assess the effects of flavors stratified by age and gender.

## Conclusions

While there is a misconception that flavors are relatively safe due to their perpetual consumption in the food, inhalation of flavors may have deleterious effects on several organ systems. The studies presented in this review have focused on the detrimental systemic effects of flavors and their flavoring agents, vanillin, menthol, and aromatic aldehydes, such as benzaldehyde, cinnamaldehyde, on the heart, lung, brain, and other organs. There is an imperative need to conduct studies in in vivo models and randomized controlled trials to understand the effect that flavors have on heart rate variability, heart and lung development, physiology and pathology, their permeability through the blood-brain barrier, and consequent sensorimotor and behavioral changes. Such studies will help design future public health guidelines regarding flavored e-C exposure.
